# The effect of osteopontin and osteopontin-derived peptides on preterm brain injury

**DOI:** 10.1186/s12974-014-0197-0

**Published:** 2014-12-03

**Authors:** Anna-Maj Albertsson, Xiaoli Zhang, Jianmei Leavenworth, Dan Bi, Syam Nair, Lili Qiao, Henrik Hagberg, Carina Mallard, Harvey Cantor, Xiaoyang Wang

**Affiliations:** Perinatal Center, Department of Neuroscience and Physiology, Sahlgrenska Academy at University of Gothenburg, Box 432, SE-405 30 Gothenburg, Sweden; Department of Pediatrics, The Third Affiliated Hospital of Zhengzhou University, 7 Kangfu Front St, 450052 Zhengzhou, China; Department of Cancer, Immunology and AIDS, Dana-Farber Cancer Institute, Harvard Medical School, 1 Jimmy Fund Way, Boston, MA 02115 USA; Department of Microbiology and Immunobiology, Division of Immunology, Harvard Medical School, 77 Ave Louis Pasteur, Boston, MA 02115 USA; Department of Pediatrics, Song Jiang Central Hospital, 746 Songjiang Zhongshan West Rd, 201600 Shanghai, China; Perinatal Center, Department of Obstetrics and Gynecology, Sahlgrenska Academy at University of Gothenburg, Journalvägen 6, 41685, Gothenburg, Sweden; Department of Perinatal Imaging and Health, Division of Imaging Sciences and Biomedical Engineering, King’s College London, King’s Health Partners, St. Thomas’ Hospital, London, SE1 7EH UK

**Keywords:** Preterm, Brain injury, Osteopontin, Hypoxia, Ischemia

## Abstract

**Background:**

Osteopontin (OPN) is a highly phosphorylated sialoprotein and a soluble cytokine that is widely expressed in a variety of tissues, including the brain. OPN and OPN-derived peptides have been suggested to have potential neuroprotective effects against ischemic brain injury, but their role in preterm brain injury is unknown.

**Methods:**

We used a hypoxia-ischemia (HI)-induced preterm brain injury model in postnatal day 5 mice. OPN and OPN-derived peptides were given intracerebroventricularly and intranasally before HI. Brain injury was evaluated at 7 days after the insults.

**Results:**

There was a significant increase in endogenous OPN mRNA and OPN protein in the mouse brain after the induction of HI at postnatal day 5. Administration of full-length OPN protein and thrombin-cleaved OPN did not affect preterm brain injury. This was demonstrated with both intracerebroventricular and intranasal administration of OPN as well as in OPN-deficient mice. Interestingly, both N134–153 and C154–198 OPN-derived peptides increased the severity of brain injury in this HI-induced preterm brain injury model.

**Conclusions:**

The neuroprotective effects of OPN are age-dependent, and, in contrast to the more mature brain, OPN-derived peptides potentiate injury in postnatal day 5 mice. Intranasal administration is an efficient way of delivering drugs to the central nervous system (CNS) in neonatal mice and is likely to be an easy and noninvasive method of drug delivery to the CNS in preterm infants.

## Background

Preterm brain injury, especially in combination with very low birth weight, has become the dominant form of brain injury in neonates. These brain injuries are associated with impaired quality of life due to resulting disorders such as cerebral palsy and behavioral, social, attentional and cognitive deficits. Preterm brain injury is generally thought to consist primarily of white-matter injury that is accompanied by elements of gray-matter injury and is characterized by a loss of premyelinating oligodendrocytes (pre-OLs) and a deficiency in their development into myelinating oligodendroglia [1]. In infants with preterm brain injury, especially preterm infants with very low birth weight, the abnormal growth and maturation of susceptible cell types, particularly oligodendrocytes and neurons, are associated with decreased cerebral and cerebellar volumes and increases in cerebral ventricular size [2].

Osteopontin (OPN) is a glycoprotein with an arginine–glycine–aspartic acid (RGD) motif that binds to the α_v_β_1_, α_v_β_3_, α_v_β_5_, α_v_β_6_, α_8_β_1_ and α_5_β_1_ integrin receptors. It is widely expressed in a variety of tissues, including the brain. OPN is also highly expressed in injured tissues and has been proposed to enhance wound healing by modulating inflammation and fibrosis and by promoting Th17 and Th1 responses [3,4]. OPN is cleaved posttranslationally by matrix metalloproteinases and thrombin into an N-terminal fragment and a C-terminal fragment, and the two cleavage products are thought to retain biological activity [5].

In mouse models of stroke, administration of full-length OPN, thrombin-cleaved OPN and peptides based on sequences from the N-terminal and C-terminal sides of the thrombin cleavage site all protect against ischemic brain injury [6,7]. In the neonatal brain, OPN is the most highly expressed gene in a postnatal day 9 (PND9) hypoxia-ischemia (HI)-induced neonatal brain injury mouse model [8], and strong OPN immunoreactivity has been seen in axons at the periphery of the ischemic zone in both subacute and chronic brain injury lesions in human newborn infants [9]. OPN enhances endogenous brain injury repair and protects the neonatal brain from HI-induced brain injury in PND9 mice [10] and PND7 rats [11], both of which are animal models that correspond to neonatal hypoxic-ischemic encephalopathy (HIE) in near-term human infants. All of this evidence suggests that OPN and OPN-derived peptides might represent promising candidates for neuroprotection. However, it is unknown how they contribute to *preterm* brain injuries. Many characteristics of rodents before PND7—such as the maturation and development of oligodendrocytes [12] and antigen-presenting dendritic cells in the immune system [13] and the expression of OPN receptor α_v_β_1_ integrin on oligodendrocytes [14]—are similar to those seen in human brain development at preterm ages [12]. This indicates that the degree of neuroprotection mediated by OPN might be very different in PND5 rodents compared to older rodents. The aim of the present study was to investigate the role of OPN and OPN-derived peptides in a mouse model of preterm brain injury.

## Methods

### Animals

C57BL/6J mice deficient in OPN (OPN^−/−^; B6.129S6(Cg)-*Spp1*^*tm1Blh*^/J) and matched wild-type control mice were purchased from The Jackson Laboratory (Bar Harbor, ME, USA) and were bred in the animal facility at the University of Gothenburg (Experimental Biomedicine, University of Gothenburg). The mice were housed with a 12:12-hour light–dark cycle and had free access to a standard laboratory chow diet (B&K, Solna, Sweden) and drinking water. All animal experiments were approved by the Animal Ethical Committee of the University of Gothenburg (no. 5/2013).

### Hypoxia-ischemia procedure

At PND5, wild-type mice and OPN^−/−^ mice of both sexes were subjected to HI insult according to a method described previously [15-17]. Briefly, mice were anesthetized with isoflurane (5.0% for induction and 1.5% to 3.0% for maintenance) in a 1:1 mixture of nitrous oxide and oxygen. The left common carotid artery was ligated, and the mice were returned to their cages and allowed to recover for 1 hour. The mice were then placed in an incubator perfused with a humidified gas mixture (10% ± 0.01% oxygen in nitrogen) at 36°C for 70 minutes. The combination of arterial ligation and hypoxia resulted in injury only in the hemisphere ipsilateral to the arterial ligation (the left hemisphere), and no injury was produced in the contralateral hemisphere (the right hemisphere). After HI-induced injury, the pups were returned to their dams until they were killed at 6 hours, 3 days or 7 days after HI-induced injury.

### Drugs

#### Recombinant mouse osteopontin

Recombinant mouse OPN (rmOPN, glycosylated) was purchased from Sigma-Aldrich (O2260; Sigma-Aldrich, St Louis, MO, USA) and reconstituted in phosphate-buffered saline (PBS).

#### Preparation of thrombin-cleaved osteopontin

**Figure 1:**
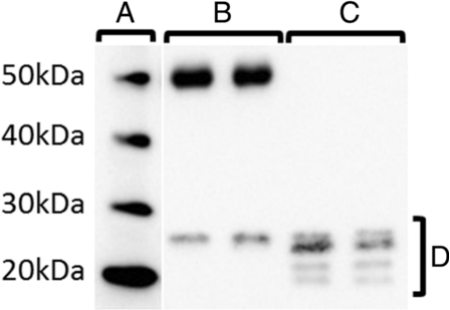
**A representative Western blot shows the efficiency of thrombin cleavage of recombinant mouse osteopontin. (A)** Molecular weight marker. **(B)** Untreated recombinant mouse osteopontin (rmOPN) protein. **(C)** Thrombin-treated rmOPN protein. **(D)** Cleavage fragments of thrombin-treated OPN.

Thrombin cleavage of rmOPN was performed using a Thrombin CleanCleave Kit (RECOMT; Sigma-Aldrich) following the instructions from the manufacturer with some modifications. Briefly, 50 μl of thrombin-agarose resin was centrifuged for 5 minutes at 500 × *g* to pellet the thrombin-agarose resin. The supernatant was removed, and the thrombin-agarose resin was resuspended and washed two times in 250 μl of 1× cleavage buffer, followed by centrifugation at 500 × *g* for 5 minutes. The supernatant was removed and discarded. After the second wash, the thrombin-agarose resin was resuspended in 50 μl of 10× cleavage buffer, 400 μl of rmOPN (200 μg/ml in PBS) was added and the mixture was incubated for 72 hours at 37°C in a shaking water bath. After incubation, the resin was removed by centrifuging the mixture for 5 minutes at 500 × *g* and collecting the supernatant. To further purify the thrombin-cleaved OPN (T-OPN), the supernatant was centrifuged twice at 10,000 × *g* for 5 minutes. After each centrifugation, the supernatant was collected and any remaining thrombin-agarose resin was discarded. The same procedure was performed for preparation of the vehicle, but 400 μl of PBS was added to the 10× cleavage buffer and thrombin-agarose resin mixture instead of 400 μl of rmOPN. The efficiency of the thrombin cleavage is shown in the Western blots in Figure 1.

#### Peptides

OPN-derived peptides from the sequences on the N-terminal and C-terminal sides of the thrombin cleavage site in the OPN protein were custom synthesized by New England Peptide (Gardener, MA, USA). The peptide from the N-terminal fragment was synthesized from OPN amino acids 134 to 153 (N134-153), and the peptide from the C-terminal fragment was synthesized from OPN amino acids 154 to 198 (C154-198). The peptide sequences were as follows:

(N134-153): IVPTVDVPNGRGDSLAYGLR

(C154-198): SKSRSFQVSDEQYPDATDEDLTSHMKSGESKESLDVIPVAQLLSM

### Drug administration

#### Intracerebroventricular injection

rmOPN, N134–153 and C154–198 were dissolved in PBS and were injected into the left lateral ventricle immediately before HI in PND5 mice. Control mice received injections of vehicle alone (PBS). The pups were anesthetized with isoflurane (5.0% for induction and 1.5% to 3.0% for maintenance), and 2 μl of drug or vehicle was injected using a syringe attached to a microinjection pump (CMA Microdialysis, Stockholm, Sweden) at a speed of 1.25 μl/min at a depth of 1.9 mm from the skull skin surface. Table 1 shows the doses and concentrations of rmOPN, N134–153 and C154–198 used for intracerebroventricular (ICV) injections.

**Table 1:** **Doses used for drug administration** ^**a**^

	**Intracerebroventricular administration**			**Intranasal administration**		
	**rmOPN**	**N134–153**	**C154–198**	**rmOPN**	**T-OPN**	**N134–153**
**Dose (μg/pup)**	0.05	0.2	0.5	1.2	1.2	30
**Concentration (μg/μl)**	0.025	0.1	0.25	0.2	0.2	5
**Volume (μl)**	2	2	2	6	6	6

^a^C154–198, Osteopontin C-terminal residues 154 to 198; N134–153, Osteopontin N-terminal residues 134 to 153; rmOPN, Recombinant mouse osteopontin; T-OPN, Thrombin-cleaved osteopontin.

#### Intranasal administration of recombinant mouse osteopontin and osteopontin-derived peptides

For all intranasal administrations, PND5 pups were laid on their backs and held gently to prevent them from moving during administration. One hour before intranasal administration, the pups were pretreated with hyaluronidase to improve the delivery of protein/peptide (total of 60 U in 6 μl, H-4272; Sigma-Aldrich, Gothenburg, Sweden) [18]. The hyaluronidase was administered by applying a single 1.5-μl drop (10 U/μl) to each nostril of the pup, and, when the drop had been inhaled by the pup, a second drop was added to each nostril.

Immediately before and after the pups were put under 10% oxygen (hypoxia), rmOPN, T-OPN, N134–153 or vehicle was administered to the pups by applying a 1.5-μl drop to each nostril. After the drop was inhaled by the pup, a second drop was administered. The doses and concentrations used in each treatment are shown in Table 1.

The rmOPN protein and N134–153 peptide used for intranasal administration were both reconstituted in PBS. Thus, PBS served as the vehicle for both treatments, and the results from the vehicle treatment in both groups were pooled when the data were analyzed. For T-OPN, PBS treated with the Thrombin CleanCleave Kit was used as the vehicle.

### Immunohistochemical staining

At 6 hours, 3 days or 7 days after HI-induced injury, the mice were deeply anesthetized and perfused intracardially with saline and 6% buffered formaldehyde (Histofix; Histolab, Gothenburg, Sweden). Their brains were dissected out, paraffin-embedded and cut into 10-μm coronal sections.

Antigen recovery was performed by heating the sections in 10 mM boiling sodium citrate buffer (pH 6.0) for 10 minutes. Nonspecific binding was blocked for 30 minutes with 4% horse, goat or donkey serum (depending on the species used to raise the secondary antibody) in PBS. The primary antibodies used were mouse anti-MAP2 (1:2,000 dilution, microtubule-associated protein 2 antibody; Sigma-Aldrich), mouse anti-MBP (1:10,000 dilution, myelin basic protein, SMI 94; BioLegend, Dedham, MA, USA) and rabbit anti-OPN (1:400; Immuno-Biological Laboratories, Gunma, Japan). After incubating the brain sections with the primary antibodies overnight at 4°C, the appropriate biotinylated secondary antibodies (1:250; all from Vector Laboratories, Burlingame, CA, USA) were added for 60 minutes at room temperature. Visualization was performed using the Vectastain ABC Elite Kit (Vector Laboratories) with 0.5 mg/ml 3,3′-diaminobenzidine enhanced with 15 mg/ml ammonium nickel sulfate, 2 mg/ml β-D-glucose, 0.4 mg/ml ammonium chloride and 0.01 mg/ml β-glucose oxidase (all from Sigma-Aldrich).

For immunofluorescent staining, sections were incubated with the following primary antibodies: rabbit anti-OPN (1:400; Immuno-Biological Laboratories), mouse anti-Iba-1 (1:750; EMD Millipore, Billerica, MA, USA), mouse anti-GFAP (1:50; Sigma-Aldrich) and mouse anti-NeuN (1:50; EMD Millipore) at 4°C overnight. After primary antibody incubation, the sections were incubated with appropriate combinations of secondary antibodies, including donkey anti-rabbit Alexa Fluor 488 (1:500), donkey anti-rabbit Alexa Fluor 594 (1:500) and donkey anti-mouse Alexa Fluor 488 (1:500; all from Molecular Probes, Leiden, the Netherlands), for 1 hour at room temperature, and a coverslip was mounted using ProLong Gold antifade reagent with 4′,6-diamidino-2-phenylindole (DAPI, Life Technologies, Carlsbad, CA, USA).

### Assessment of white-matter/gray-matter injury

The extent of white-matter/gray-matter injury was evaluated at 7 days after HI-induced injury. For evaluation of brain injury, every 50th section throughout the brain, or a representative section from the hippocampal level, was stained for the presence of microtubule-associated protein 2 (MAP2) or myelin basic protein (MBP).

Gray-matter injury was quantified by measuring the total area of MAP2-positive staining at 4× magnification using the Micro Image version 4.0 software package (Micro-Macro AB, Gothenburg, Sweden). White-matter injury was quantified by measuring the area of MBP-positive staining [19,20]. Tissue loss was calculated by subtracting the amount of staining in the ipsilateral hemisphere from that of the contralateral hemisphere.

### RT-PCR

OPN expression in the brain at 6 hours, 24 hours and 7 days after HI-induced injury at PND5, as well as in uninjured control mice, was analyzed by RT-PCR.

For PCR, the mice were deeply anesthetized and perfused intracardially with saline at 6 hours, 24 hours or 7 days post-HI, and the brains were rapidly dissected out, divided into ipsilateral and contralateral hemispheres, snap-frozen and stored at −80°C until use. Both the ipsilateral and contralateral hemispheres from HI-injured mice were used, and only the left hemispheres from uninjured control mice were used.

A QIAzol Lysis Reagent homogenizer (Qiagen, Solna, Sweden) was used to homogenize the brain tissue, and total RNA was extracted using the RNeasy Lipid Tissue Mini Kit (Qiagen). RNA levels were measured on a spectrophotometer at 260-nm absorbance. The QuantiTect Reverse Transcription Kit (Qiagen) was used to synthesize cDNA according to the manufacturer’s instructions. The primers used were OPN (spp1) (QT00157724) and 18S ribosomal RNA (QT01036875) (both from Qiagen).

Each reaction was run in duplicate, and the amplification protocol was run on a LightCycler 480 apparatus (Roche, Stockholm, Sweden). Melting curves were analyzed to ensure that only one PCR product had been produced. A standard curve using increasing concentrations of cDNA was generated for quantification and for estimating amplification efficiency. The amplification transcripts were quantified with the relative standard curve and normalized to the 18S ribosomal RNA reference gene.

### Western blot analysis

Total protein from brain homogenate was analyzed by Western blotting to determine the endogenous expression of OPN in the brain during normal development. Mice were killed by decapitation at PND3, PND9, PND21 or PND60, and their brains were rapidly dissected out, divided into two hemispheres and snap-frozen. The brains were homogenized by sonication in ice-cold, RNase-free PBS buffer containing 2% protease inhibitor cocktail (P8340; Sigma-Aldrich) and 10 mM ethylenediaminetetraacetic acid and stored at −80°C until use.

Samples of brain homogenate were denatured in gel-loading buffer at 70°C for 10 minutes and loaded onto a 4–12% Tris-Bis gel (Life Technologies). Proteins were transferred to a nitrocellulose membrane (0.2 μm, Optitran; Schleicher & Schuell, Dassel, Germany). After blocking with 30 mmol/L Tris-HCl (pH 7.5), 100 mmol/L NaCl and 0.1% Tween 20 containing 5% fat-free milk powder for 1 hour at room temperature, the membranes were incubated with polyclonal anti-OPN primary antibody (1:2,000, ab8448; Abcam, Cambridge, UK) for 1 hour at room temperature, followed by incubation with goat anti-rabbit horseradish peroxidase–labeled secondary antibody (1:5,000; Vector Laboratories) for 1 hour at room temperature. SuperSignal Western Dura chemiluminescent substrate (Thermo Scientific, Waltham, MA, USA) and a LAS-1000 cooled charge-coupled device camera (Fujifilm, Tokyo, Japan) were used for visualization, and Image Gauge software (Fujifilm) was used for quantification of immunoreactive bands.

### Statistics

The SPSS software package v19.0 (SPSS, Chicago, IL, USA) was used for all analyses. Data were expressed as mean ± standard deviation (SD). Comparisons between groups were performed using Student’s *t*-test, and data with unequal variance were compared with the Mann–Whitney *U* test. Analysis of variance followed by the least significant difference *post hoc* test was used for comparison of data from more than two groups. *P*-values <0.05 were considered statistically significant.

## Results

### Endogenous osteopontin expression in the neonatal mouse brain

**Figure 2:**
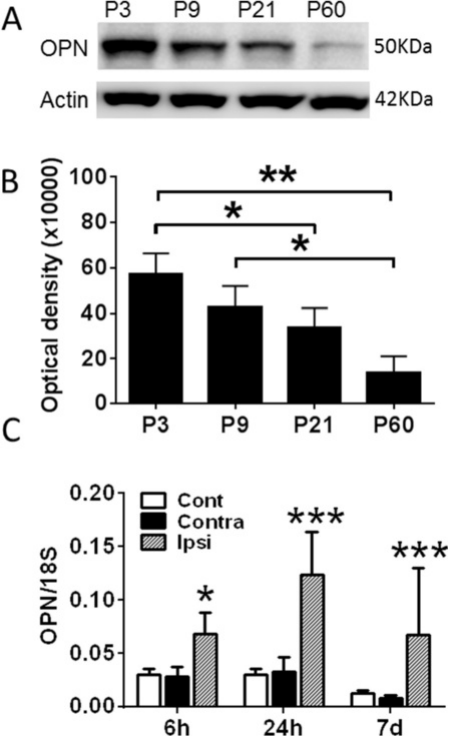
**Endogenous expression of osteopontin in the brain in normal and hypoxia-ischemia-injured mice.** In uninjured mice, the expression of endogenous osteopontin (OPN) during development was analyzed by Western blotting using the whole-brain homogenates at postnatal day 3 (P3), P9, P21 and P60. **(A)** A representative Western blot. **(B)** The quantification of the Western blot analysis by measuring the density of the immunoreactive bands (*n* = 3). **(C)** The mRNA expression of endogenous OPN in the left hemisphere of uninjured control mice (Cont) and the contralateral (Contra) and ipsilateral (Ipsi) hemispheres after hypoxia-ischemia (HI) injury (*n* = 6 to 8). The data are presented as the ratios between OPN and reference gene (18S) mRNA expression. No changes in the 18S mRNA expression were observed in the brain after HI-induced injury. Data presented are the mean ± SD. *P < 0.05; **P < 0.01; ***P < 0.001.

We first examined the endogenous expression of OPN in wild-type mice during normal development. Western blot analysis of total protein in whole-brain homogenate from normal uninjured mice at PND3, PND9, PND21 and PND60 showed that OPN expression in the brain decreased with age. At PND9, an age that is comparable to that of human near-term infants, the expression of OPN in the brain was already starting to decrease compared to PND3 mice (Figures 2A and B).

To evaluate whether the expression of OPN was regulated after HI-induced injury in PND5 mice, brains were harvested at 6 hours, 24 hours or 7 days postinjury and total RNA was analyzed by RT-PCR. OPN mRNA expression was significantly increased in the brain hemisphere ipsilateral to the ligated carotid artery compared to the contralateral hemisphere and compared to uninjured mice at 6 hours, 24 hours and 7 days after HI insult (Figure 2C). There was no difference in the contralateral hemisphere of the treated animals compared to those that had not been exposed to HI (Figure 2C).

**Figure 3:**
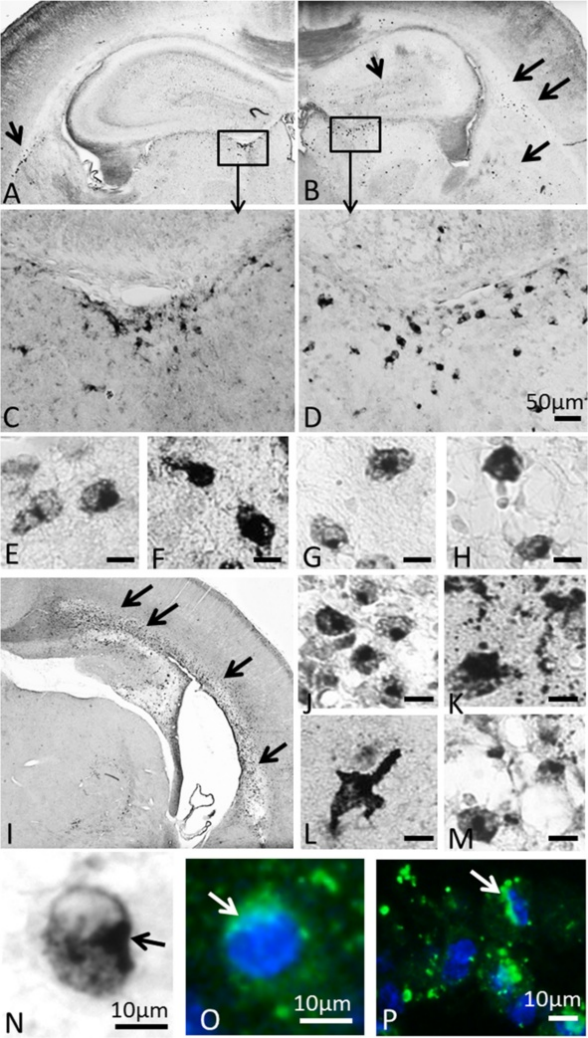
**Increased expression of OPN protein in the neonatal mouse brain after hypoxia-ischemia-induced brain injury. (A)** through **(H)** Osteopontin (OPN)-positive staining (arrows in **A** and **B**) in the contralateral hemisphere **(A and C)** and ipsilateral hemisphere **(B and D**) at 6 hours after hypoxia-ischemia (HI)-induced injury. The positive staining was seen in the hippocampus, the lateral ventricle area **(C and D)**, the thalamus and the meninges. **(C)** and **(D)** Enlarged images of the periventricular areas shown within the boxed areas in **(A)** and **(B)**, **(E)** through **(H)** Higher-magnification images of OPN-positive cells from the periventricular region **(E and F**) and white matter **(G and H)** of the ipsilateral hemisphere. **(I)** through **(M)** At 3 days after HI, OPN-positive immunostaining was found mainly in the subcortical white matter area (arrows in **I**) in the ipsilateral hemisphere. **(J)** through **(M)** Higher-magnification images of OPN-positive cells in the ipsilateral hemisphere white matter **(J)**, thalamus **(K)** and cortex **(L and M)**. **(N)** through **(P)** The perinuclear expression of OPN (arrows) in the brain after HI. Green: OPN staining. Blue: nuclear staining. Bars in**D** = 50 μm; bars in **E** through **H**, **J** through **M**, and **P** = 10 μm; bars in **N** and **O** = 10 μm.

To confirm the expression of OPN protein in the brain after HI, immunohistochemistry using an anti-OPN antibody was performed on paraffin-embedded brain sections from 6 hours or 3 days after HI-induced injury (Figure 3). At 6 hours after HI, OPN-positive staining in both the contralateral (Figures 3A and C) and the ipsilateral (Figures 3B and D) hemispheres was observed in the periventricular areas (Figures 3C and D), the hippocampus, the white matter, the thalamus and the meninges. Significantly greater staining was seen in the ipsilateral hemisphere (Figures 3B,D to H) compared to the contralateral hemisphere (Figures 3A and C). At 3 days post-HI, the OPN-positive staining was located mainly in the ipsilateral subcortical white matter (Figures 3I and J), thalamus (Figures 3I and K), hippocampus (Figure 3I) and the part of the cortex adjacent to the subcortical white matter (Figures 3I,L and M). Interestingly, we noticed that OPN-positive cells showed a perinuclear distribution of the OPN protein in the cells (Figures 3N, O and P) that was similar to the distribution of intracellular OPN seen in a previous study [21].

**Figure 4:**
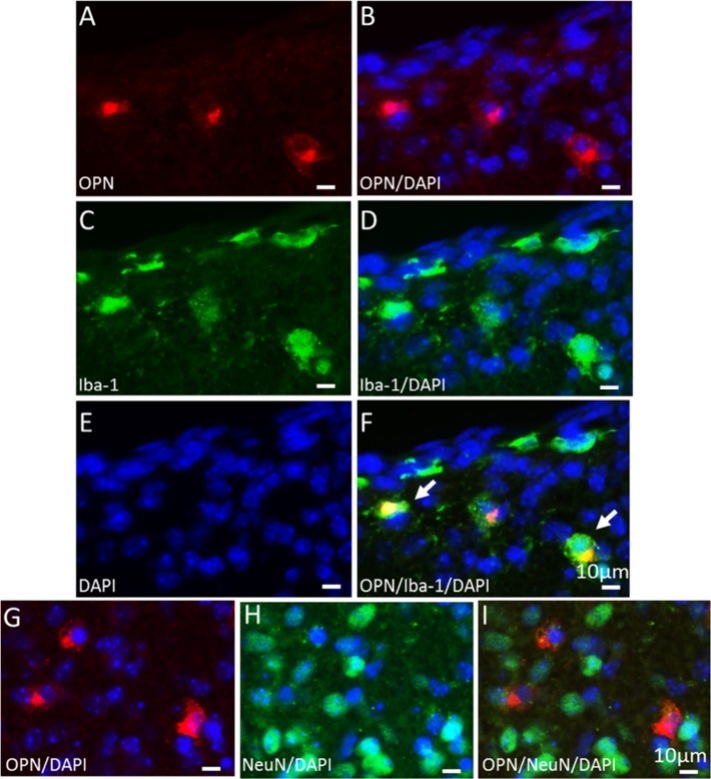
**Osteopontin expression in microglia, astrocytes and neurons in the neonatal mouse brain after hypoxia-ischemia-induced injury.** Representative pictures of immunofluorescent double staining show the costaining of osteopontin (OPN) (red in **A**, **B** and **F**) with Iba-1 (green in **C**, **D** and **F**) and with NeuN (green in **H** and **I**) in the periventricular area in the ipsilateral hemisphere at 6 hours after HI. Arrows in **(F)** indicate cells that coexpress OPN and Iba-1. DAPI (blue in **B**, **D**, **E**-**I**).

Immunofluorescent double labeling showed that the majority of the OPN-positive cells were double-labeled with the microglial marker Iba-1 (Figures 4A to F), none colocalized with the neuronal marker NeuN (Figures 4G to I) and only a few colocalized with the astrocyte marker glial fibrillary acidic protein (GFAP, data not shown).

### Effect of T-OPN-, rmOPN- and OPN-derived peptides on HI-induced preterm brain injury in postnatal day 5 mice

Intranasal administration is a noninvasive method of drug delivery that can bypass the blood–brain barrier and provide therapeutic substances with direct access to the central nervous system (CNS), and this route of administration of drugs and cells is also efficient in the immature brain injury rodent models [22,23]. Especially, intranasal delivery has been shown to be an efficient way of delivering rmOPN protein and peptides to the CNS after carefully evaluating their penetrance, spreading and persistence in the brain through intranasal administration [6], and rmOPN and T-OPN have been shown to provide efficient protection against ischemic brain injury in a mouse model of stroke [6]. Therefore, we sought to determine if rmOPN and T-OPN also confer neuroprotection in a preterm brain injury model.

**Figure 5:**
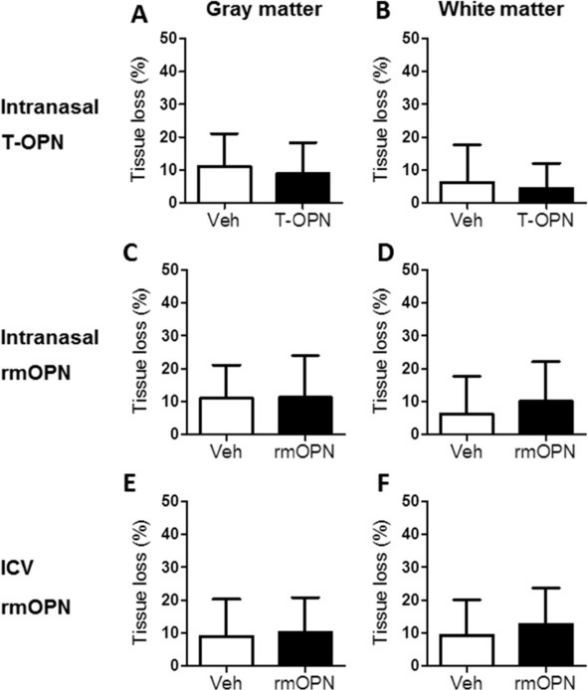
**The effect of thrombin-cleaved osteopontin and recombinant mouse osteopontin on preterm brain injury.** Bar graphs show the total tissue volume loss (%) in the gray matter **(A, C and E)** and white matter **(B, D and F)** of mice given intranasal thrombin-cleaved osteopontin (T-OPN) **(A and B)**, intranasal recombinant mouse OPN (rmOPN) **(C and D)** and intracerebroventricular (ICV) rmOPN **(E and F)** at 7 days after hypoxia-ischemia (HI)-induced injury. Intranasal administration: vehicle (Veh), *n* = 20; T-OPN, *n* = 14; rmOPN, *n* = 17. ICV administration: vehicle (Veh), *n* = 31; rmOPN, *n* = 27. Data presented are the mean ± SD.

T-OPN was administered intranasally immediately before and after hypoxia in an HI-induced preterm brain injury model in PND5 mice. The efficiency of thrombin cleavage of rmOPN was analyzed by Western blotting, which showed that all of the rmOPN had been successfully cleaved by thrombin (Figure 1). At 7 days after induction of HI, the brain injury in the gray matter and white matter was evaluated by immunostaining of MAP2 and MBP, respectively. However, no difference was seen between the T-OPN-treated group and the vehicle-treated group (Figures 5A and B) in terms of either gray-matter (Figure 5A) or white-matter (Figure 5B) injury. Similarly, we did not observe any significant differences in gray- or white-matter injury after intranasal rmOPN treatment (Figures 5C and D).

To evaluate the possibility that the lack of effect was due to technical issues with the intranasal administration, we next administered rmOPN using ICV injection. ICV injection is a well-accepted method for the delivery of drugs directly to the CNS in rodent models that we have successfully used in our previous studies [24,25], and ICV administration of exogenous OPN has been shown to protect both adult mice [7] and neonatal rats [11] from ischemic brain injury. When we compared the tissue loss in gray matter and white matter between the rmOPN-treated animals and the vehicle-treated animals, we detected no difference between the rmOPN-treated group and the control mice in either the gray matter (Figure 5E) or the white matter (Figure 5F), and these findings corresponded well to the results from the intranasal administration (Figure 5C, D).

**Figure 6:**
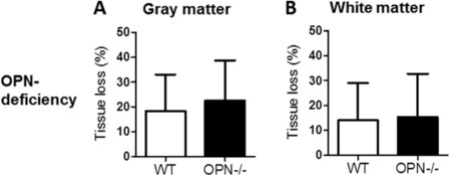
**The effect of osteopontin deficiency on preterm brain injury.** Bar graphs show the total tissue volume loss (%) in the gray matter **(A)** and white matter **(B)** of wild-type (WT) and osteopontin (OPN)-deficient (OPN^−/−^) mice at 7 days after hypoxia-ischemia-induced brain injury. WT: *n* = 13; OPN^−/−^: *n* = 15. Data presented are mean ± SD.

To further evaluate the effect of rmOPN on HI-induced preterm brain injury, OPN^−/−^ animals were used. The lack of protective effect of rmOPN was further confirmed by ICV administration of rmOPN or vehicle immediately before hypoxia in OPN^−/−^ mice (data not shown). Furthermore, the volume of HI-induced gray-matter (Figure 6A) and white-matter (Figure 6B) tissue loss was not different comparing OPN^−/−^ and wild-type mice, and this suggests that endogenous OPN does not affect development of brain injury in the preterm brain.

**Figure 7:**
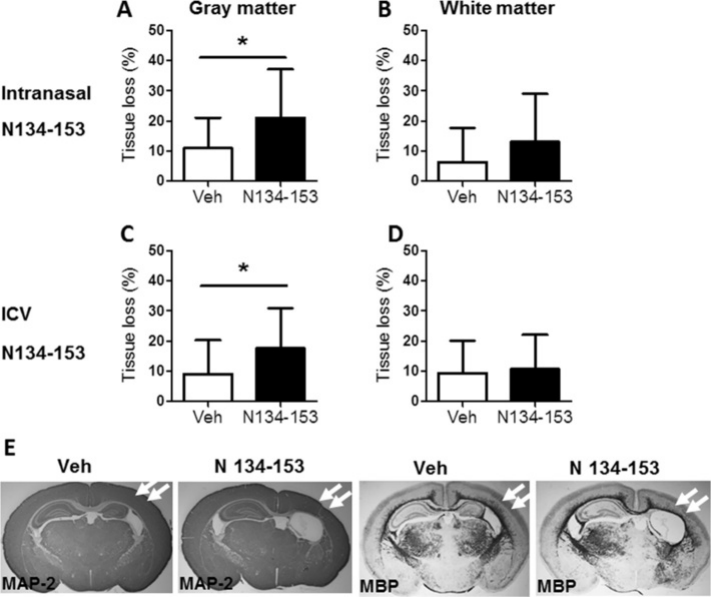
**The effect of the osteopontin-derived N134–153 peptide on preterm brain injury.** Bar graphs show the total tissue volume loss (%) in the gray matter **(A and C)** and white matter **(B and D)** in mice that received intranasally administered osteopontin amino acids 134 to 153 (N134–153) **(A and B)** and intracerebroventricularly (ICV) administered N134–153 **(C and D)** at 7 days after hypoxia-ischemia (HI)-induced injury. Intranasal administration: vehicle (Veh), *n* = 20; N134–153, *n* = 16. ICV administration: vehicle (Veh), *n* = 31; N134–153, *n* = 15. Data presented are the mean ± SD. *P < 0.05. **(E)** Representative immunohistochemical stains of microtubule-associated protein 2 (MAP-2) (gray matter) and myelin basic protein (MBP) (white matter) showing injury (arrows) in brain sections of the vehicle-treated (Veh) and N134–153-treated mice at 7 days after HI injury in postnatal day 5 mice.

In an adult mouse model of stroke, intranasal administration of the OPN-derived N134–153 peptide provided significant neuroprotection [6]. To examine whether intranasal administration of this short peptide could also provide neuroprotection in our HI-induced preterm brain injury model, we administered the OPN-derived N134–153 peptide, which contains the RGD motif, to neonatal mice immediately before and after hypoxia at PND5. Surprisingly, intranasal administration of the N134–153 peptide resulted in exacerbated injury with significantly increased tissue loss of gray matter, but not of white matter, compared to vehicle at 7 days after HI (Figures 7A and B). To make sure that the peptide really had this effect in the brain and that this unexpected increase in injury severity was not an experimental artefact attributable to the intranasal delivery, we also administered the peptide using ICV administration. We found that ICV administration of N134–153 worsened the HI-induced gray-matter brain injury in the PND5 mouse model (Figure 7C) but had no effect on the white-matter injury (Figure 7D).

**Figure 8:**
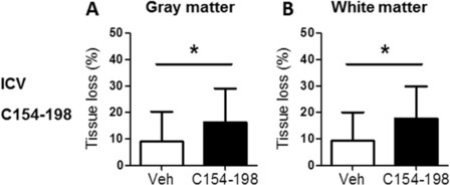
**The effect of the C154–198 peptide on preterm brain injury.** Bar graphs show the total tissue volume loss (%) in the gray matter **(A)** and white matter **(B)** of the intracerebroventricularly (ICV) treated C154–198 peptide mouse group at 7 days after hypoxia-ischemia (HI)-induced injury. Vehicle (Veh), *n* = 31; C154–198, *n* = 23. Data presented are the mean ± SD. *P < 0.05.

With these unexpected results showing increased brain injury with the N134–153 peptide, we used a second peptide, the C154–198 peptide, generated from the sequence at the C-terminal fragment of the OPN protein after thrombin cleavage, which has also been shown to be protective in an adult stroke model [6]. Because we had shown that the intranasal administration and the “traditional” ICV delivery methods gave similar results for rmOPN and N134–153 in our model, C154–198 was administered only by the ICV route. Again, mice treated with C154–198 displayed increased injury in the gray matter (Figure 8A), as well as significantly increased tissue loss in the white matter, compared to the vehicle-treated control group (Figure 8B).

## Discussion

In this study, we evaluated the effect of the exogenous OPN protein as well as OPN-derived peptides on the outcome of HI-induced preterm brain injury in mice. We found that the OPN-derived N134–153 and C154–198 peptides exacerbated the brain injury, but neither full-length rmOPN nor T-OPN had any effect on HI-induced preterm brain injury. This lack of effect of full-length OPN was seen after administration of rmOPN to both wild-type mice and OPN^−/−^ mice subjected to HI and also in OPN^−/−^ mice subjected to HI alone. We also found that intranasal delivery of N134–153 to PND5 mice before HI had an effect on brain injury comparable to ICV administration, which suggests that intranasal administration is an efficient way of delivering drugs to the CNS in mouse models of HI-induced preterm brain injury.

There is evidence that OPN plays an important role in the pathogenesis of adult white-matter injury diseases such as multiple sclerosis, as well as in animal models of experimental autoimmune encephalomyelitis (EAE). Increased OPN expression is frequently found in the spinal cord in patients with multiple sclerosis [26-28] and at the injury site in animal models of white-matter disease [29,30]. OPN^−/−^ mice are resistant to progressive EAE [26,31], and OPN is upregulated during *in vivo* demyelination and remyelination and enhances myelin formation *in vitro* [29]. Taken together, these findings suggest that OPN might play a detrimental role in multiple sclerosis, at least in the inflammatory phase.

In some other ischemic brain injury cases, such as in the adult rodent models, OPN^−/−^ mice exhibited unaltered infarction volume [7,32] but greatly increased retrograde degeneration in the thalamus in a stroke model [32]. Administration of rmOPN protein [7] or administration of a short peptide containing the RGD motif resulted in significantly reduced infarction size in the brain [6]. However, OPN enhanced endogenous repair and was protective in mouse and rat models of HI-induced neonatal brain injury [10,11].

Although we found increased expression of OPN in the microglia after HI in the mouse preterm brain injury model, which is in agreement with previous findings [8,11], we did not find any protective effect of rmOPN against HI-induced preterm brain injury. This lack of effect was further confirmed using OPN^−/−^ mice. These results differ from previous studies in which researchers showed that rmOPN protects PND7 rats from HI-induced brain injury [11] and that OPN^−/−^ mice sustain more severe injury in a PND9 HI brain injury mouse model [10]. These models correspond to the brain injury seen in near-term human infants. A power analysis was performed to make sure that the differing results in the present study compared to previous studies were not related to an issue of using an insufficient number of animals.

Inflammation stimulates both pro- and anti-inflammatory processes, and the balance of these processes can be either beneficial or harmful depending on what other inputs the cell is receiving. It is known that full-term infants with HIE and animal models of “near-term” brain injury typically exhibit cortical and mixed deep gray-matter injury [33], whereas diffuse white-matter injury, together with gray-matter abnormalities, are the most common types of cerebral abnormalities associated with prematurity [2,34-36]. Apart from differences in brain injury pathology, previous studies have indicated that the cell death mechanisms in brain injury differ at different developmental stages [37,38], and it has been found that the expression of some integrin units varies during different developmental stages. For example, the migratory and proliferating precursor oligodendrocytes express α_v_β_1_, but postmigratory and postmitotic differentiated oligodendrocytes upregulate α_v_β_5_ and α_v_β_1_ disappears. These integrin subunits have a functional role in regulating the proliferation and differentiation in oligodendrocyte progenitor cells [39] and in myelination [40-42], and they affect the sensitivity of oligodendrocytes to the survival effect of growth factors [43]. It is known that pre-OLs, which are vulnerable cell types in preterm brain injury, are expressed most abundantly in PND2 to PND5 rodents and quickly decline in older rodents [12]. By PND7, extensive oligodendrocyte maturation occurs in both mice and rats and coincides with the onset of early myelination [44].

Integrin subunits, including the α_v_ and β_2_ integrin subunits that act as OPN receptors, are important regulators during the development of oligodendrocytes [14], cortical neurons [45-47], postnatal synaptogenesis [48] and synaptic function [49]. Moreover, the distribution of integrin subunits is different between the white and gray matter at an early postnatal age [50]. Thus, it is possible that the different integrin expression levels at different oligodendrocyte/neuronal development stages control the delicate binding between the integrins and their ligands and lead to different outcomes. These various aspects might partly explain the different effects of OPN on brain injury in our preterm brain injury model using PND5 mice compared to the effects of OPN in the neonatal near-term brain injury model using PND9 mice [10] and PND7 rats [11] and in the adult stroke model [6,7].

A molecular dissection of OPN [4,51-54] has defined two functionally distinct domains of the OPN protein. The N-terminal fragment of T-OPN contains the RGD tripeptide that interacts with several α_v_-containing integrins [3,55-57]. This interaction mediates the antiapoptotic effects of OPN through several pathways, including inhibition of inducible nitric oxide synthase activity [58,59]. The C-terminal fragment of T-OPN interacts with CD44 receptors and promotes Th17-dependent inflammatory responses and subsequent white-matter injury [51,54,60,61]. These results indicate that the N- and C-terminal fragments of T-OPN might have different effects on the progression of preterm brain injury. In the present study, both the RGD-containing N134–153 peptide and the C154–198 peptide worsened brain injury in the HI-induced preterm brain injury model. The difference in effects of the N134–153 and C154–198 peptides is that the N134–153 peptide mainly affected the gray matter, whereas the C154–198 peptide had an effect on both the gray and white matter. One explanation for this distinction is that the two peptides interact with different receptors using different binding sites. The N134–153 peptide contains the RGD motif that is known to bind specific integrin receptors, such as α_v_β_1_, α_v_β_3_, α_v_β_5_, α_v_β_6_, α_8_β_1_ and α_5_β_1_, whereas the C154–198 peptide fragment does not contain this RGD motif and binds to other receptors, such as CD44. Our results indicate that binding sites from the N- and C-terminal fragments of OPN after thrombin cleavage, rather than the full-length OPN protein, might be used to further explore possible therapeutic strategies for preterm brain injury. However, the exact site of action and the underlying mechanisms of the peptide in preterm brain injury need to be further explored.

Intranasal delivery of high-molecular-weight biologics such as proteins, gene vectors and stem cells is a potentially useful strategy to treat a variety of diseases and disorders of the CNS, including stroke, and is an easy and noninvasive method of drug delivery [62] that has shown promise in several clinical trials [63]. The rationale for intranasal delivery stems from the unique anatomy of the olfactory region of the nasal cavity. Specialized olfactory receptor cells in this region are the major “open windows” to the CNS and allow drugs to bypass the blood–brain barrier. Accumulating evidence indicates that a wide range of substances, including high-molecular-weight substances, water-soluble molecules, nucleic acids, viruses and even whole cells can enter the CNS of many species, including humans, after intranasal administration [64].

Intranasal administration can deliver OPN to the area of the brain that is rendered ischemic by middle cerebral artery occlusion (MCAO), and intranasal administration of T-OPN protects the adult mouse brain from MCAO injury [6]. It has previously been shown that intranasal administration is also a valid method for delivery of cells to the CNS in newborn mice [65]. In the present study, we found that intranasal delivery of N134–153 had the same effect as ICV administration. These results show for the first time that intranasal delivery can serve as an efficient noninvasive method for drug delivery to the preterm brain and suggest that intranasal delivery could be explored as a simple and efficient method of delivering drugs to the CNS in preterm infants with brain injury.

## Conclusions

We found that OPN expression increased after induction of HI in the preterm brain injury model. Administration of rmOPN and T-OPN did not confer any protective effect, but both the OPN-derived RGD-containing N134–153 and the C154–198 peptides increased the severity of the brain injury. This indicates that the neuroprotective effects of OPN are age-dependent and that, in contrast to the more mature brain, OPN-derived peptides potentiate injury in PND5 mice.

## Abbreviations

HI: Hypoxia-ischemia
HIE: Hypoxic-ischemic encephalopathy
ICV: Intracerebroventricular
MAP2: Microtubule-associated protein 2
MBP: Myelin basic protein
OPN: Osteopontin
PBS: Phosphate-buffered saline
PND: Postnatal day
pre-OL: Premyelinating oligodendrocyte
RGD: Arg-Gly-Asp tripeptide
rmOPN: Recombinant mouse osteopontin
T-OPN: Thrombin-cleaved osteopontin
